# Splenic Trauma in the Immunocompromised: Unveiling Complexities and Dilemmas

**DOI:** 10.7759/cureus.60718

**Published:** 2024-05-20

**Authors:** Bala Manohar, Jaspreet Shergill, Harmandeep S Jabbal, Divakar Goyal, Mahendra P Singh

**Affiliations:** 1 General Surgery, All India Institute of Medical Sciences, Bathinda, Bathinda, IND; 2 Trauma and Emergency, All India Institute of Medical Sciences, Bathinda, Bathinda, IND

**Keywords:** vaccination policy, prophylaxis, overwhelming post-splenectomy infections, splenectomy, splenic trauma

## Abstract

The incidence of splenectomy due to traumatic injuries has decreased globally, owing to the advancements in hospital facilities and angioembolization techniques. Nevertheless, some patients still undergo splenectomy, leading to a lifelong risk of post-splenectomy sepsis. This risk is particularly heightened in immunocompromised individuals, presenting significant challenges in managing and preventing such infections. Compounding these challenges is the absence of comprehensive national guidelines and a splenic registry. While there have been improvements in postoperative prophylaxis through vaccination, patient education, and antibiotic usage, evidence supporting these strategies in immunocompromised patients remains lacking. Thus, there is an urgent need for expanded research in these areas to mitigate the morbidity and mortality associated with post-splenectomy sepsis in this vulnerable population. We report our experience of a young male having a penetrating abdominal injury who underwent splenectomy and had an immunocompromised status with both Human Immunodeficiency Virus (HIV) and Hepatitis C Virus (HCV) positive status.

## Introduction

The spleen, a crucial lymphoid organ, plays a pivotal role in both innate and adaptive immunity, safeguarding the body against invading pathogens [1]. While splenectomy for hematological and malignant conditions persists, its necessity for traumatic reasons has declined in recent years due to advancements in non-operative management and the availability of angioembolization [2].

The removal of the spleen exposes individuals to heightened risks of infections, particularly from encapsulated organisms and intraerythrocytic parasites. Among these complications, overwhelming post-splenectomy infections stand out with a mortality rate exceeding 50% [3]. This risk escalates further in immunocompromised individuals, necessitating a protocol-based management approach.

Presented herein is a case of an immunocompromised patient presenting with penetrating abdominal injury, resulting in grade 4 splenic injury, who underwent splenectomy.

## Case presentation

**Figure 1:**
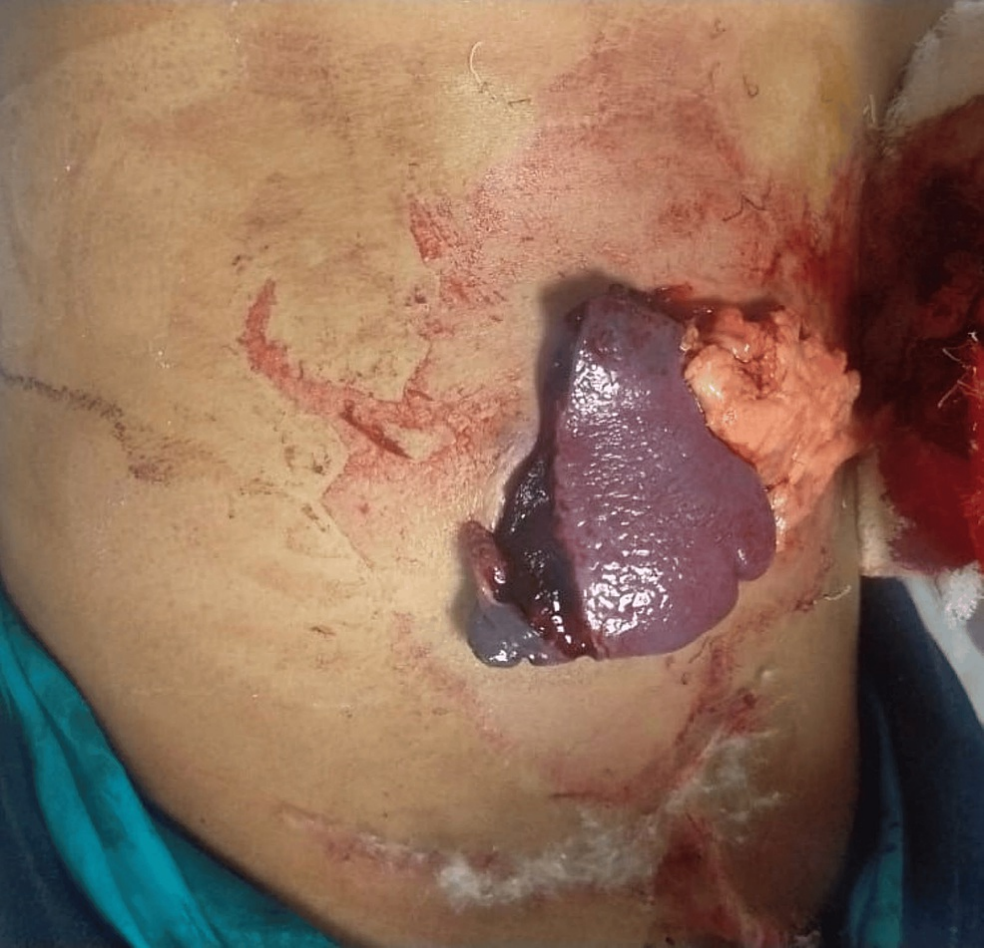
Injury over the abdomen with splenic evisceration

A 27-year-old male patient presented to the emergency department after being struck by a bull, resulting in a penetrating abdominal injury with the spleen protruding from the abdominal wall (Figure 1)

The patient exhibited grade 4 hemorrhagic shock. Extended Focused Assessment Ultrasonography for Trauma was positive for all abdominal quadrants and left hemithorax. Immediate resuscitation commenced following the Advanced Trauma Life Support (ATLS) protocol, which included endotracheal intubation and placement of a left-sided intercostal drainage (ICD) tube to address a hemothorax with a subsequent observation of 400 ml of blood and air gush post-ICD placement. Blood gas analysis revealed metabolic acidosis with respiratory alkalosis and despite resuscitation efforts, the patient's condition deteriorated, necessitating immediate transfer to the operating room.

**Figure 2:**
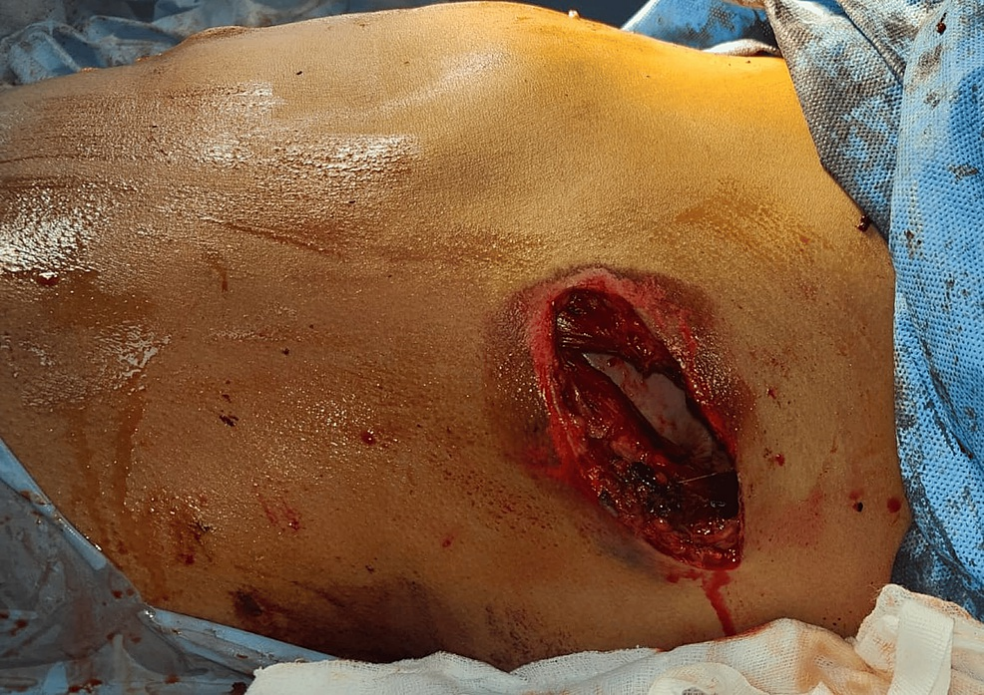
Penetrating wound over the abdominal wall

**Figure 3:**
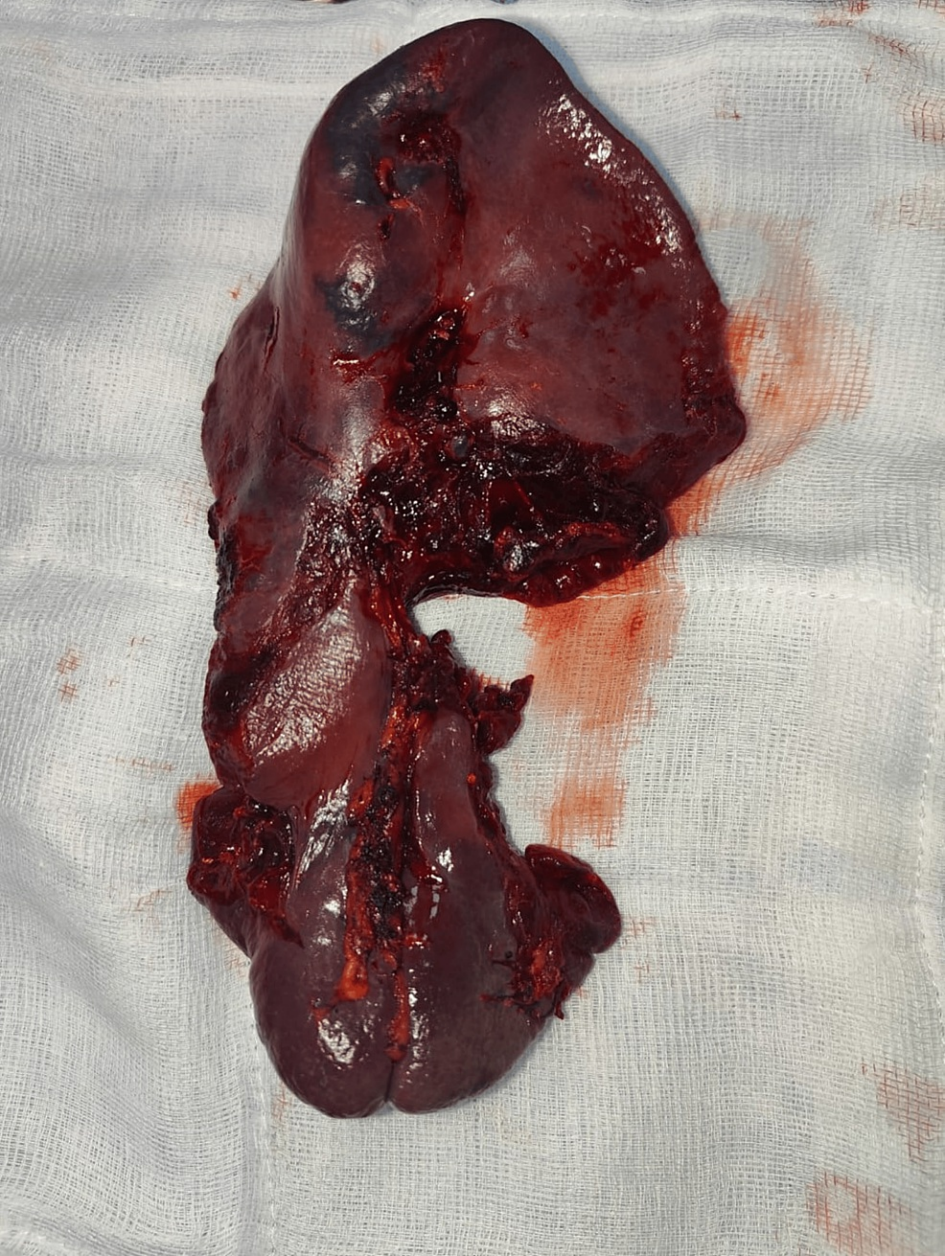
Shattered Spleen

Upon opening the abdomen, approximately 1500 ml of blood loss was noted, and the spleen was found to be completely shattered. Additionally, the patient had sustained a diaphragmatic injury with a fracture of the 11th rib. Splenectomy was performed alongside repair of the diaphragmatic rent, and the patient was subsequently transferred to the Surgical Intensive Care Unit (ICU) for further management (Figures 2, 3).

Given the patient's history of intravenous drug abuse by his wife, he experienced withdrawal symptoms, complicating the weaning process. The tracheostomy was performed on postoperative day 5, and once the patient stabilized, he was transferred to the general ward. Gradual reduction of oxygen support was initiated, leading to successful decannulation of tracheostomy.

Subsequent blood investigations revealed that the patient was positive for both Human Immunodeficiency Virus (HIV) and Hepatitis C Virus (HCV) infection, prompting further evaluation with Hepatitis C Virus Ribonucleic Acid (HCV RNA) and Cluster of Differentiation 4 (CD4) count tests, yielding values of 1,00,000 IU/litre and 150 cells per cubic millilitre, respectively. The patient was given with pneumococcal vaccine, meningococcal vaccine, and *Haemophilus influenzae *type b (Hib) vaccine on postoperative day 14. He was treated with amoxicillin-clavulanic acid for 15 days. Rigorous precautions were taken to prevent chest and surgical site infections, and the patient was discharged on oral amoxicillin-clavulanic acid therapy after suture removal. Follow-up after one week revealed the patient to be asymptomatic.

## Discussion

Approximately 9.75 out of 10,000 individuals in the general population usually undergo splenectomy [4]. Among these individuals, there is a lifetime risk of 5% of developing overwhelming post-splenectomy infection (OPSI), characterised by fulminant sepsis or meningitis with septic shock and disseminated intravascular coagulation [5-6]. Mortality rates of up to 50% have been documented in patients experiencing OPSI, underscoring the critical importance of preventing this fatal complication [3].

The Centers for Disease Control and Prevention (CDC) has issued recommendations outlining a list of vaccines and their corresponding schedules for patients undergoing splenectomy [7]. Despite these guidelines, numerous studies in the literature highlight the prevalent issue of inadequate adherence to proper vaccination protocols among these individuals.

Kealey et al studied the vaccination trends in patients undergoing splenectomy following trauma. Their findings revealed that initial vaccination rates stood at 76%, 75%, and 68% for *Streptococcus pneumoniae*, *Neisseria meningitidis*, and *Haemophilus influenzae *type b (Hib), respectively. However, the rates of revaccination were notably lower, with only 39% and 15% for *S. pneumoniae *and *N. meningitidis*, respectively [8].

Boam et al observed adherence to vaccination recommendations following splenectomy for all indications. Their findings indicated that 91.5% of patients received peri-operative vaccinations for Hib, meningococcus C, and pneumococcus, while 84% received booster doses for pneumococcus. Additionally, 95% of patients received annual influenza vaccinations [9].

In India, a study conducted at a prestigious institution, Jawaharlal Institute of Postgraduate Medical Education and Research (JIPMER), in 2021 by Sureshkumar et al underscored the insufficient knowledge regarding prophylaxis among surgical residents, alongside the significant barriers of high costs and limited availability of vaccines, contributing to suboptimal vaccine coverage [10].

Furthermore, there are no national guidelines addressing post-splenectomy patients, and globally, specific protocols for double immunocompromised individuals remain absent. The absence of a spleen registry exacerbates these challenges, complicating patient management and care decision-making.

There is a notable absence of evidence-based guidelines regarding antibiotic prophylaxis for these patients in the literature [11]. In individuals with double immunocompromised status, the significance of antibiotic prophylaxis and its duration is heightened. Given the dynamic nature of the disease and its impact on the effectiveness of antibiotics and vaccines, it is crucial to develop tailored guidelines for these vulnerable patients.

## Conclusions

In conclusion, the prevention and management of post-splenectomy sepsis present numerous challenges, exacerbated by factors such as limited awareness among surgical residents and the general population, high costs of vaccines, and their unavailability in government healthcare facilities. Compounded by the prevalence of immunocompromised patients from low socioeconomic backgrounds, particularly in countries like India, the hurdles are further heightened. Moreover, ensuring adequate follow-up care adds another layer of complexity to patient management. To address these multifaceted challenges, we propose the establishment of a nationwide spleen registry coupled with standardised guidelines for the prevention of post-splenectomy complications. Such initiatives hold promise in enhancing awareness, accessibility, and continuity of care for individuals at risk, ultimately improving patient outcomes and reducing the burden of post-splenectomy sepsis.
